# Economic Burden of Alzheimer Disease and Related Dementias by Race and Ethnicity, 2020 to 2060

**DOI:** 10.1001/jamanetworkopen.2025.13931

**Published:** 2025-06-05

**Authors:** Stipica Mudrazija, María P. Aranda, Darrell J. Gaskin, Stephanie Monroe, Patrick Richard

**Affiliations:** 1 Health Systems and Population Health, School of Public Health, University of Washington, Seattle; 2 Income and Benefits Policy Center, Urban Institute, Washington, DC; 3 Edward R. Roybal Institute on Aging, Suzanne Dworak-Peck School of Social Work, University of Southern California, Los Angeles; 4 Johns Hopkins Center for Health Disparities, Health Policy and Management Bloomberg School of Public Health, Johns Hopkins University, Baltimore, Maryland; 5 Health Equity, UsAgainstAlzheimer’s, Washington, DC; 6 Health Services Administration, Preventive Medicine and Biostatistics, Uniformed Services University, Bethesda, Maryland

## Abstract

**Importance:**

Alzheimer disease and related dementias (ADRD) have substantial clinical and public health consequences for individuals, families, employers, and government.

**Objective:**

To assess ADRD’s economic burden on non-Latino African American, Latino, and non-Latino White adults and their caregivers, employers, and the government between 2020 and 2060.

**Design, Setting, and Participants:**

Population-based cross-sectional study using nationally representative data on African American, Latino, and White adults aged 50 years and older with ADRD and their unpaid caregivers from the 2014 to 2020 Medical Expenditure Panel Survey (MEPS) alongside the 2011 to 2017 National Study of Caregiving (NSOC) and 2013 Panel Study of Income Dynamics. These data were augmented with information from the US Census Bureau, Bureau of Labor Statistics, Internal Revenue Service, and other sources to estimate current and future economic burden. Two-part regression models were used to estimate medical and work-related costs for older adults, and multivariate-distance matching was used to estimate the value of unpaid care, lost wages and productivity, loss of federal income tax revenue, and financial transfers for caregivers. Data were analyzed from March 2023 to February 2025.

**Exposure:**

Older adults with ADRD and their family caregivers.

**Main Outcomes and Measures:**

Projected medical costs and work-related losses for persons with ADRD, and unpaid care value, forgone earnings, and lost federal income tax payments and labor productivity for caregivers.

**Results:**

Of 31 028 older adults in MEPS, 5184 (10%) were African American; 146 (<1%) American Indian or Alaska Native; 1043 (3%) Asian (Indian, Chinese, or Filipino); 5346 (10%) Latino; 690 (2%) Other Asian, Native Hawaiian, and Pacific Islander; and 18 617 (75%) were White. In the NSOC sample of 1929 older adults, there were 644 (33%) African American, 169 (9%) Latino, and 1116 (58%) White adults. The total estimated economic burden of ADRD was close to $344 billion in 2020 and was projected to increase to over $3 trillion in 2060. African American and Latino adults bore one-third ($113 billion) of it in 2020, with projections rising to $1.7 trillion by 2060, surpassing the economic burden for White adults, which was projected to grow from $231 billion to $1.4 trillion.

**Conclusions and Relevance:**

The findings of this study suggest that African American and Latino older adults with ADRD and their families are likely to face disproportionately high burdens, primarily associated with unpaid caregiving. Understanding ADRD prevalence, comorbidity, inadequate care, and support policies may attenuate economic burdens for all US residents.

## Key Points

**Question:**

What is the current and future economic burden of Alzheimer disease and related dementias (ADRD) for non-Latino African Americans, Latino, and non-Latino White adults through 2060?

**Findings:**

This cross-sectional study of 31 028 older adults found that the estimated total economic burden of ADRD for African American and Latino adults was $113 billion in 2020 and was projected to reach $1.7 trillion by 2060 (in nominal dollars), surpassing the economic burden for White adults, which was projected to grow from $231 billion to $1.4 trillion. African American and Latino caregivers to adults with ADRD were estimated to be more adversely financially burdened by care provision than White caregivers, and this difference was projected to grow further in the coming decades.

**Meaning:**

These findings suggest that African American and Latino adults with ADRD and their caregivers face high and fast-growing adverse burdens of ADRD, primarily for unpaid care and forgone wages.

## Teaser

This cross-sectional study estimates the economic burden of Alzheimer disease and related dementias on non-Latino African American, Latino, and non-Latino White adults, their caregivers, employers and the government between 2020 and 2060.

## Introduction

Alzheimer disease is the most common form of dementia, affecting an estimated 6.9 million US residents aged 65 and older.^1^ Other forms of dementia include cerebrovascular disease, frontotemporal degeneration, Lewy body disease, hippocampal sclerosis, and Parkinson disease, among others. Jointly termed Alzheimer Disease and Alzheimer Disease-Related Dementias (AD/ADRD, herein ADRD), these are debilitating neurocognitive disorders that impair memory, thought processes, and functioning, resulting in disability and ultimately death.^1^ People living with ADRD require ongoing health care and long-term services and supports (LTSS) due to cognitive, behavioral, affective, social, and financial changes experienced over time.^1,2^ At advanced stage, individuals typically require substantial hands-on care, have significant mobility and self-care limitations, and become high utilizers of health care and LTSS, including palliative and institutionalized care.^3^ Family members provide most hands-on caregiving, many with substantial consequences to their own health and well-being, such as depression, anxiety, poor sleep, decreased social activities, and poor health.^4^

ADRD is one of the costliest diseases in the US. One recent estimate suggests that the total cost of ADRD-related caregiving is $305 to $450 billion,^5^ while another suggests that the ADRD-related cost of unpaid care is almost $347 billion and the cost of formal care $360 billion.^1^ These are substantially higher costs than for cancer ($21.1 billion),^6^ stroke ($56.5 billion),^7^ heart disease ($239.9 billion),^8^ or even diabetes ($412.9 billion).^9^ ADRD increases medical care costs in 2 ways: the costs of treating ADRD, and the costs of treating other health conditions for persons living with ADRD.^7^ Those participating in the labor market incur costs due to lost wages. ADRD impacts family caregivers through time spent providing unpaid care and limiting ability to work.^10^ Even when working, they experience lower productivity.^11^ Some family caregivers also provide financial support (ie, make financial transfers) to persons living with ADRD, adversely affecting their savings and expenditures.^12^ Government is also negatively impacted through the loss of income tax revenue and possibly increased costs for social programs. Finally, many persons living with ADRD need professional care in institutional settings or the community.

African American and Latino adults likely face disproportionate economic burden of ADRD. Existing evidence points to differences in biological and nonbiological processes that could lead to such an outcome, including high prevalence rates and associated comorbidities (hypertension, diabetes, and obesity), less access and lower quality education, and low access to quality health care, including lack of specialists and minoritized health care practitioners in general.^1,13,14,15,16,17^ African American and Latino adults also have low access to evidenced-based treatments, interventions, and caregivers’ support.^18^ They earn less and accumulate less wealth than others.^19^

Building on previous estimates,^20,21^ this study assesses the economic burden of ADRD for non-Latino African American, Latino, and non-Latino White populations in the US (henceforth, we omit the non-Latino prefix) between 2020 and 2060. We hypothesize that projections will show substantial increases in the economic burden across all racial and ethnic groups due to population aging and the growing number of people living with ADRD. We further hypothesize that the economic burden will grow particularly fast for the 2 minoritized populations, consistent with the empirical evidence presented previously.

## Methods

### Data Source

To compute medical care costs and work-related losses for persons with ADRD, we used data from the 2014 to 2020 Medical Expenditure Panel Survey (MEPS), a nationally representative survey of the US civilian noninstitutionalized population.^22^ Data on the costs of formal care services through 2060 come from the Genworth’s Cost of Care Survey, on nursing home populations from the Kaiser Family Foundation, and on the racial and ethnic composition of nursing home population from the US Centers for Disease Control and Prevention. To assess the value of unpaid care and associated work-related costs for caregivers of persons with ADRD, we used data on family caregivers from the 2011 to 2017 National Study of Caregiving (NSOC), a nationally representative supplement to the National Health and Aging Trends Study. We match these caregivers to noncaregivers from the 2013 Panel Study of Income Dynamics (PSID), a nationally representative longitudinal panel survey of US families. Additional data for projections come from the US Census Bureau, US Bureau of Labor Statistics, and the Internal Revenue Service. All these data are publicly available and deidentified, and our study was exempt from institutional review and the need for informed consent in accordance with 45 CFR §46. This study followed Strengthening the Reporting of Observational Studies in Epidemiology (STROBE) reporting guidelines for cross-sectional studies.^23^

### Study Population

Our pooled MEPS sample had 31 028 adults aged 50 and over. The pooled NSOC sample included 1929 unpaid family caregivers to Medicare beneficiaries aged 65 years and older, matched to 8615 similar noncaregivers from PSID. We distinguish subsamples of African American, Latino, and White adults across the 3 surveys, with respondents self-reporting race and ethnicity information.

### Study Outcomes

For adults living with ADRD, we computed excess medical care spending, lost wages and labor market productivity, and lost income tax. We also approximated costs of formal care, including nursing homes and paid home care. For their unpaid caregivers, we computed the value of the unpaid care (ie, replacement cost of caregiving), forgone wages (ie, opportunity cost of caregiving), lost labor market productivity, financial transfers to care recipients, and lost federal income tax. We refer to these costs as the economic burden of ADRD.

### Statistical Analysis

Medical care spending includes payments for hospital-based services, such as inpatient, outpatient, and emergency services; prescription drugs; and other services. These payments include health plan payments and out-of-pocket costs. Work-related loss includes forgone wages and disability days. We used the incremental costs technique that estimates differences between persons with and without ADRD adjusting for demographic, socioeconomic, and health factors.^24,25,26,27^ We employed 2-part regression models using the survey regression procedures that incorporate complex design factors and sample weights.^28,29,30^

In the analysis of caregivers of persons with ADRD, we computed the value of their unpaid care, lost wages, lost productivity, lost federal income tax revenue, and financial transfers to care recipients using multivariate-distance matching of caregivers with noncaregivers. To derive future estimates, we used the Census Bureau’s National Population Projections. We repeated the caregiver-noncaregiver matching by college degree attainment for the 3 major racial and ethnic groups to accommodate for the historic trend of educational attainment growth and divergence in real wages by education.^31^ We established future real wage growth trajectory by educational attainment using the means observed in the 2 decades preceding the COVID-19 pandemic.

Approximations of future costs of formal care use the same demographic information alongside the existing projections of nursing home and home paid care costs through 2060, the information on the share of older adults in the community who receive paid care, and the racial and ethnic composition of nursing home population. While our approximations did not allow us to establish formal estimates of uncertainty, we assessed the sensitivity of our results to varying assumptions. For example, we calculated the value of caregiving using 2 different wage valuations and examined the possibility of no increase in nursing home population. While these calculations do not directly introduce bias, they reflect any underlying bias in the existing estimates, such as race and ethnicity-specific ADRD prevalence rates.^32^

Results are reported in 2020 dollars. Following the recommendations by the Second Panel on Cost-Effectiveness in Health and Medicine, we used a 3% annual rate of increase to adjust the estimates of future ADRD economic burdens.^33^ In Table 1, we provide more information on data sources^34,35,36,37,38,39,40,41,42,43,44,45^ and estimation approach by outcomes. Detailed methodological information is in the eMethods in Supplement 1. Data were analyzed from March 2023 to February 2025.

**Table 1.** Overview of Outcome Measures, Data Sources, and Approaches to Current and Future Estimates^a^

Outcome measure	Data source	Approach
Care recipients		
Medical care costs and work-related losses	MEPS, 2014-2020 (N = 31 028 and 3458 for medical care and work hours, respectively)^34^; National Population Projections, 2018^35^; R-CPI-U-RS, 2023^36^	Present estimates are from a 2-part regression model on a pooled MEPS sample that includes the following covariates: age, race and ethnicity, gender, education, income, marital status, health insurance status, self-reported health, and various health conditions (ADRD, diabetes, asthma, asthma attack, cancer, high blood pressure, heart attack, angina, other heart disease, emphysema/chronic bronchitis, joint pain, and arthritis. Future estimates use Census Bureau’s detailed population projects by age and race and ethnicity alongside CPI index for base year value adjustment and assume 3% annual growth in nominal values from the base year.
Paid home care and nursing home care	Genworth Cost of Care Survey, 2023^36^; Chidambaram and Burns, 2024^37^; NCHS, 2019^38^; Reckrey et al, 2020^39^	We use existing cost projections from the Genworth Cost of Care Survey through 2060 (which apply the same 3% inflation factor that we use in other calculations). These data are supplemented with the current and historic information on nursing home population from the Kaiser Family Foundation (Chidambaram and Burns, 2024), as well as with data on the share of nursing home population by race and ethnicity from the CDC (NCHS, 2019), and information on the prevalence and intensity of paid community care is from Reckrey et al, 2020.
Caregivers		
Caregiving value (ie, replacement cost of care)	NSOC, 2011-2017 (N = 1929)^40^; Bureau of Labor Statistics, 2023^41^; R-CPI-U-RS, 2023^36^; National Population Projections, 2018^35^	We use a pooled sample of caregivers to Medicare beneficiaries aged 65 years and older from the NHATS to assess the current number of caregivers in the US and their mean hours of care by race and ethnicity. We calculate the value of this care using Bureau of Labor Statistics data on mean hourly wages for health and personal care aides’ and national minimum wage, respectively, for 2 alternative replacement cost valuations. Consumer Price Index retroactive series (R-CPI-U-RS) information from the Bureau of Labor Statistics (2023) is used to adjust estimates to US dollars in the base year. Finally, National Population Projections are used to project future (nominal) value of caregiving assuming 3% annual inflation rate from the base year.
Forgone wages (ie, opportunity cost of care), lost labor productivity, and caregivers’ financial transfers to care recipients	NSOC, 2011-2017 (N = 1929)^40^; PSID, 2013 (N = 8615)^42^; CPS, 2023^43^; R-CPI-U-RS, 2023^36^; National Population Projections, 2018^35^	We perform multivariate-distance matching of caregivers from NSOC with noncaregivers from PSID, stratifying the matching by race and ethnicity and requesting exact match with respect to caregivers’ gender and marital status. The samples are matched on: age, gender, marital status, education attainment, homeownership, self-rated health status, and any living siblings. The matching is done for the full sample and stratified by college degree attainment and race and ethnicity. Historical trends in educational attainment, household income, and mean wages by race and ethnicity from the CPS are used to extrapolate their future trends. As previously, CPI data are used to adjust dollar values to the base year and National Population Projections to project future values of these caregiving-related costs assuming 3% annual inflation from the base year.
Lost federal income tax payments	NSOC, 2011-2017 (N = 1929)^40^; PSID, 2013 (N = 8615)^42^; CPS, 2023^43^; R-CPI-U-RS, 2023^36^; National Population Projections, 2018^35^; IRS, 2022^44^	In addition to information provided in the prior box, we use data on federal income tax payments by adjusted gross income levels for tax year 2020 (filed in 2021) from the IRS to approximate lost federal income tax payments.

Abbreviations: CDC, US Centers for Disease Control and Prevention; CPI, Consumer Price Index; CPS, Current Population Survey; IRS, Internal Revenue Service; MEPS, Medical Expenditure Panel Survey; NCHS, National Center for Health Statistics; NHATS, National Health and Aging Trends Study; NSOC, National Study of Caregiving; PSID, Panel Study of Income Dynamics; R-CPI-U-RS, CPI for all Urban Consumers.

^a^ Information on sample sizes is included only for microdata sources that we use in our calculations. This information is not available for summary estimates from government and other publicly available sources of data that are based on the underlying microdata collected and/or analyzed by those entities (eg, population estimates from the US Census Bureau).

## Results

### Older Adults Living With ADRD

Of 31 028 older adults in MEPS, 5184 (10%) were African American; 146 (<1%) American Indian or Alaska Native; 1043 (3%) Asian (Indian, Chinese, or Filipino); 5346 (10%) Latino; 690 (2%) Other Asian, Native Hawaiian, and Pacific Islander; and 18 617 (75%) were White. In the NSOC sample of 1929 there were 644 (33%) African American, 169 (9%) Latino, and 1116 (58%) White adults. In our MEPS sample, almost half of respondents were 65 and older, with a similar share of males and females and those with at least some college education. African American and Latino population each accounted for about 10 percent of the sample. For additional descriptive information, see eTable 1 in Supplement 1.

The total estimated economic burden of ADRD was close to $344 billion in 2020 and was projected to increase to over $3 trillion in 2060. ADRD-related medical care spending was almost $20 billion in 2020 (Table 2). Minoritized populations accounted for almost 20% ($4 billion), split roughly equally between African American and Latino adults (Table 1). These costs were projected to reach $147 billion in 2060, with faster growth for Latino ($32 billion) and African American adults ($24 billion) than White adults ($91 billion). The work-related costs for adults with ADRD were over $4 billion in 2020, and were projected to surpass $22 billion in 2060, with African American and Latino adults’ share increasing from 24% to 43%. The economic burden of ADRD for African American and Latino populations was $113 billion in 2020 and was projected to increase to $1.7 trillion in 2060, surpassing the economic burden for the White population (increase from $231 billion to $1.4 trillion).

**Table 2.** Total Annual Medical Care Costs and Labor Productivity Costs Associated With Alzheimer Disease and Related Dementias, by Race and Ethnicity

Type of cost	Cost, billions, $US				
	2020	2030	2040	2050	2060
Medical care costs					
African American	2.1	4.8	8.9	15.2	23.7
Latino	1.8	4.8	9.8	18.5	31.9
White	15.9	27.3	45.9	68.6	91.2
Work-related losses					
African American	0.5	0.9	1.5	2.2	3.5
Latino	0.5	1.1	2.2	3.7	6.0
White	3.1	4.9	6.7	9.0	12.7
Total	23.9	43.8	75.0	117.2	169.0

Actual (2020) and projected (2030, 2040, 2050, and 2060) amounts are based on population numbers from the Census Bureau; estimates are adjusted annually by 3% using 2020 as the baseline year.

### Approximation of Formal Care Costs for Persons With ADRD

In Table 3, we present an approximation of the current and future costs for nursing homes and paid home care. We assumed that the demand for these services will increase commensurately with the older population growth. However, related paid home care costs are projected to increase faster than nursing homes cost due to projected faster increase in paid care prices. Formal care costs for African Americans and Latino adults with ADRD were $19 billion and for White adults $64 billion in 2020, reaching $293 billion and $386 billion, respectively, by 2060.

**Table 3.** Current and Projected Costs of Paid Home Care and Nursing Home Care for Adults Living With Alzheimer Disease and Related Dementias Between 2020 and 2060, by Race and Ethnicity

Type of cost	Cost, billions, $US				
	2020	2030	2040	2050	2060
Paid home care^a^					
African American	3.4	9.8	17.9	30.5	48.2
Latino	3.7	12.7	26.2	48.7	84.1
White	18.7	43.1	69.4	99.9	129.4
Nursing home care^b^					
African American	8.7	20.4	37.4	63.6	100.6
Latino	3.3	9.1	18.7	34.9	60.2
White	45.6	85.3	137.4	197.8	256.2
Total	83.3	180.5	307.0	475.4	678.7

^a^ Assuming average of 10 hours of weekly care for those reporting receiving less than 20 hours of care (58%) and 30 hours of care for those reporting receiving more than 20 hours of care (42%).

^b^ Assuming three-quarters of all nursing home residents live in semiprivate and one-quarter in private rooms.

In eTable 5 in Supplement 1, we offer an alternative calculation of formal care costs that assumes no increase in nursing home population from 2020 levels, consistent with a continued push for deinstitutionalization and aging in place. Jointly, the prior estimates suggest that despite much faster expected demographic growth of minoritized populations, most public supports will continue to be for the caregiving of older White adults.

### Caregivers of Older Adults Living With ADRD

In Table 4, we provide an overview of the projected value of unpaid care for older adults living with ADRD and associated costs for caregivers, their employers, and the federal government through 2060. In 2020, the estimated value of ADRD-related unpaid care for older White adults was higher than the combined value for African American and Latino adults. By 2030, the annual combined value of care received by older African American and Latino adults was projected to surpass that received by older White adults, and the difference was projected to expand afterwards. Using home health and personal care aides’ mean wage for replacement wage valuation, the annual value of unpaid care for the 2 minoritized groups was projected to increase from just over $62 billion in 2020 to over $842 billion in 2060, of which $586 billion was attributed to Latino adults. For White adults, the value was projected to grow from $76 billion in 2020 to $382 billion in 2060.

**Table 4.** Value of Unpaid Care for Older Adults Living With Alzheimer Disease and Related Dementias and Associated Costs for Their Caregivers, Caregivers’ Employers, and the Federal Government Between 2020 and 2060, by Race and Ethnicity^a^

Type of cost	Cost, billions, US $				
	2020	2030	2040	2050	2060
Caregiving value, minimum wage					
African American	13.1	25.6	46.8	79.2	124.0
Latino	19.7	43.8	89.4	165.7	284.3
White	40.1	66.3	104.8	147.5	185.3
Caregiving value, replacement wage					
African American	24.8	49.6	92.5	159.9	255.8
Latino	37.5	84.8	176.8	334.7	586.4
White	76.1	128.4	207.4	298.0	382.1
Forgone wages, total					
African American	7.3	15.7	31.9	60.1	104.7
Latino	12.1	29.7	68.1	141.6	271.3
White	44.3	80.5	141.9	222.8	312.3
Lower labor force participation					
African American	5.8	12.4	25.3	47.5	82.6
Latino	9.4	23.3	53.9	112.9	217.8
White	34.0	61.9	109.2	171.5	240.7
Less than full-time work					
African American	1.5	3.3	6.7	12.6	22.1
Latino	2.7	6.4	14.2	28.7	53.6
White	10.3	18.7	32.8	51.2	71.5
Lost labor productivity					
African American	1.8	3.8	7.7	14.5	25.1
Latino	2.6	6.0	12.6	24.1	42.9
White	11.7	21.7	38.8	61.8	87.8
Lost federal income tax payments					
African American	0.5	1.0	2.1	3.9	6.9
Latino	0.8	1.8	4.2	8.9	17.2
White	3.1	5.7	10.2	16.2	23.0
Caregivers’ financial transfers					
African American	0.9	1.8	3.5	6.2	10.3
Latino	0.5	1.2	2.5	5.0	9.0
White	9.4	16.9	29.5	45.9	63.6

^a^ Actual (2020) and projected (2030, 2040, 2050, and 2060) amounts are based on population numbers from the Census Bureau; estimates are adjusted annually by 3% using 2020 as the baseline year.

Forgone wages for ADRD caregivers was projected to increase from $7 billion in 2020 to close to $105 billion in 2060 for African American caregivers, from $12 billion to over $271 billion for Latino caregivers, and from $44 billion to $312 billion for White caregivers. Lost wages mostly stemmed from lower labor force participation of caregivers, but approximately one-fifth was due to less intensive work among those working.

Furthermore, many unpaid ADRD caregivers report being less productive at work due to caregiving, which may adversely affect their employment and/or promotion prospects and is a cost to employers. As of 2020, this loss was higher than $4 billion for the 2 minoritized groups. By 2060, the loss was projected to increase to almost $68 billion ($43 billion for Latino caregivers), compared with $88 billion for White caregivers. Caregivers’ forgone wages and productivity losses were on a steeper growth trajectory than the value of unpaid care, suggesting that work-related losses of unpaid caregiving are becoming increasingly expensive from the society’s perspective (eFigure in Supplement 1).

Forgone wages also result in lost income tax payments on unearned income. In 2020, African American, Latino and White caregivers could have paid over $4 billion in additional federal income tax. Assuming current tax rates, by 2060 lost income tax payments were projected to reach $23 billion for White, over $17 billion for Latino, and almost $7 billion for African American ADRD caregivers.

Some unpaid caregivers also provide financial support (eg, purchasing food or medicine) to persons with ADRD. In 2020, the total value of such transfers was $11 billion. By 2060, it was projected to reach $83 billion, with more than $19 billion attributable to African American and Latino caregivers.

To get a better sense of the financial burden for individual ADRD caregivers, we next focus on trends in the per person forgone earnings and forgone earnings as a share of household income (Table 5). In 2020, Latino caregivers had the largest amount of earnings lost due to caregiving (16% of their household income) followed by African American (9%) and White (8%) caregivers. By 2050, this was projected to increase to 18%, 12%, and 9% of household income, respectively, for Latino, African American, and White caregivers. By 2060, it was projected to further increase for African American caregivers.

**Table 5.** Per Person Total Amount of Forgone Earnings and Forgone Earnings as a Share of Household Income for Caregivers of Adults Living With Alzheimer Disease and Related Dementias Between 2020 and 2060, by Race and Ethnicity^a^

Type of Cost	2020	2030	2040	2050	2060
Amount, US $					
African American	5288	7738	11 479	17 006	25 168
Latino	7376	10 921	16 369	24 467	36 488
White	6876	9936	14 471	21 090	30 757
Household income, %					
African American	9.4	10.2	11.1	12.0	12.9
Latino	15.7	16.8	17.6	17.9	17.6
White	8.1	8.5	8.9	9.0	9.0

^a^ Actual (2020) and projected (2030, 2040, 2050, and 2060) amounts are based on population numbers from the Census Bureau; estimates are adjusted annually by 3% using 2020 as the baseline year.

## Discussion

Individuals and families faced with ADRD experience economic, physical, psychological, and social burdens affecting their well-being. This article presents how ADRD is projected to affect the economic well-being of African American and Latino adults, who face the disproportionate burden of ADRD with regards to prevalence, risk factors, access to evidenced-based treatments and interventions, low quality health care, and end-of-life outcomes, and compares them with White adults.^12^ Policymakers, health care practitioners and ADRD researchers, who are developing new policies, programs, interventions, and medications should take into account the disproportionate ADRD-related economic consequences for African American and Latino adults

The economic burden of ADRD for African American and Latino populations was $113 billion in 2020 and is projected to increase to $1.7 trillion in 2060, surpassing the economic burden for the White population (increase from $231 billion to $1.4 trillion). Unpaid caregiving value accounted for about 55% of the 2020 total amount for the 2 minoritized populations, and its share will decline to 50% by 2060 as the share of work-related costs grows. Employers will also face losses in productivity, and the government will face lower tax revenue.

What are the consequences of these projected economic burdens? Individuals with ADRD and their caregivers will face an increasing financial burden resulting in the loss of wealth, including lower retirement savings, increased borrowing and debt, and increased reliance on government-funded income support and health insurance programs. As their ability to financially support their children weakens, this will lead to intergenerational transmission of inequality and long-lasting financial vulnerability for families of persons with ADRD. Given that ADRD disproportionately affects older women, and women are more likely to be caregivers than men, the financial burdens are especially pernicious for them. Weaker labor force attachment and lost labor productivity will adversely impact economic growth and public finances.

The most substantial economic burdens of ADRD are linked to unpaid care. Therefore, increased policy efforts should lessen consequences for caregivers and facilitate their dual roles as caregivers and workers. These policy interventions should recognize the needs of an increasingly diverse population, responding to unique needs and preferences of different people. This may include enhancements of programs to support employed caregivers and further expansion and flexibilization of paid family leave. Evidence suggests that paid family leave policies can support caregivers in remaining attached to the labor force, yet their impact is limited due to their current design.^46^ Examples of federal family support policies and initiatives are included in the National Family Caregiver Support Program and RAISE Family Caregivers Advisory Act,^47^ and the National Research Summit on Care, Services and Support for Persons Living with Dementia and their Care Partner/Caregivers.^48^ Other supports include expansion of community-based programs that support and compensate family members for providing care, enhanced respite programs, more in-home professional care availability, and improved coordination with family care provision. The expansion of these community-based programs should accommodate family and community settings particular to African American and Latino adults living with ADRD. Importantly, if new disease-modifying drugs are approved, medical spending (and out-of-pocket costs, if insurance coverage is limited) could substantially increase. It is unclear, however, what the net impact would be on unpaid caregiving and related costs as older adults’ cognitive health would improve, but they may also live longer with other types of disabilities. Possible decreases in public supports for older adults would have adverse financial consequences for families of persons living with ADRD, while increased prevention efforts would have the opposite effect.

Although demographic trends play a critical role in explaining the increasing share of ADRD-related economic burdens facing minoritized populations, there are other factors contributing to them being disproportionate. Delayed diagnosis and treatment, elevated rates of neuropsychiatric symptoms due to no or delayed care, comorbidities, and social determinants of health all contribute to increased ADRD rates and related burdens for minoritized individuals and their families.^49^ The issue is complicated further by the disparity in the number of minoritized physicians and specialists. For example, only 5.7% and 6.9% of the physicians in the US are African American and Latino, respectively.^16^

### Limitations

Because MEPS only includes data on noninstitutionalized persons, we had to use data from the Genworth’s Cost of Care Survey, CDC, and the Kaiser Family Foundation to approximate nursing home and paid home care costs, but we recognize the higher uncertainty of such estimates. For caregivers, we are unable to capture fully costs related to caregiver burden and their impact on caregivers’ health and well-being. Some of these impacts may be reflected in government’s increased outlays for health care and social programs, while others may be less readily quantifiable.

Our estimates do not reflect possible changes in disease progression or delayed onset due to medical and pharmacological innovations. Due to limited sample size, we disaggregate our estimates only by education and race and ethnicity. Our estimates of lost income taxes refer to federal taxes, as we lack information to assess impact on state and local taxes.

## Conclusions

Because of their higher ADRD risk and reliance on family caregiving, the economic burdens of ADRD disproportionately fall on African American and Latino populations. As these 2 populations grow and age in the coming decades, the adverse ADRD-related economic burden they experience is projected to rise substantially. In the context of health and economic equity, there is scope for policy and clinical interventions that lessen ADRD-related burdens for older Americans. As these interventions are being developed, researchers and policymakers should include African American and Latino families living with ADRD in the clinical trials and demonstration projects to improve the effectiveness of interventions for these communities.

## Notes

Supplement 1.
eMethods.
**eTable 1.** Summary Statistics for Dependents, Key Independent Variable, and Covariates Used in the Analysis **eTable 2.** Coefficients from Two-Part Models for Dependents, Key Independent Variable, and Covariates Used in the Analysis **eTable 3.** Estimated Differences in the Likelihood of Employment and Likelihood of Full-Time Employment Among Workers Between Caregivers and Matched Noncaregivers **eTable 4.** Matching Results for the Likelihood of Employment and Means and Variances of Raw and Matched Data for Noncollege Educated Dementia Caregivers and Noncaregivers **eFigure.** Ratio of Work-Related Costs to Value of Unpaid Care, 2020 to 2060 **eTable 5.** Current and Projected Costs of Paid Home Care and Nursing Home Care for Adults Living With ADRD Between 2020 and 2060 Assuming Stable Nursing Home Population at the 2020 Level, by Race and Ethnicity
eReferences.

Supplement 2.
Data Sharing Statement

## References

[1] Alzheimer’s Association. Alzheimer’s disease facts and figures. Alzheimers Dement. 2024;(Apr):30. doi:10.1002/alz.13809 PMC11095490 38689398

[2] Larson EB, Stroud C, Committee on Care Interventions for Individuals with Dementia and Their Caregivers. Meeting the challenge of caring for persons living with dementia and their care partners and caregivers: a way forward. 2021. Accessed April 29, 2025. https://nap.nationalacademies.org/catalog/26026/meeting-the-challenge-of-caring-for-persons-living-with-dementia-and-their-care-partners-and-caregivers 10.1001/jama.2021.4928 33835148

[3] Eisenmann Y, Golla H, Schmidt H, Voltz R, Perrar KM. Palliative care in advanced dementia. Front Psychiatry. 2020;11:699. doi:10.3389/fpsyt.2020.00699 32792997 PMC7394698

[4] Liang J, Aranda MP, Lloyd DA. Association between role overload and sleep disturbance among dementia caregivers: the impact of social support and social engagement. J Aging Health. 2020;32(10):1345-1354. doi:10.1177/0898264320926062 32524886

[5] Nandi A, Counts N, Bröker J, . Cost of care for Alzheimer’s disease and related dementias in the United States: 2016 to 2060. NPJ Aging. 2024;10(1):13. doi:10.1038/s41514-024-00136-6 38331952 PMC10853249

[6] Yabroff KR, Mariotto A, Tangka F, . Annual report to the nation on the status of cancer, part 2: patient economic burden associated with cancer care. J Natl Cancer Inst. 2021;113(12):1670-1682. doi:10.1093/jnci/djab192 34698839 PMC9891103

[7] US Center for Disease Control and Prevention. Stroke facts. 2023. Accessed April 29, 2025. https://www.cdc.gov/stroke/facts.htm#print

[8] US Center for Disease Control and Prevention. Heart disease facts. 2023. Accessed April 29, 2025. https://www.cdc.gov/heartdisease/facts.htm#:~:text=Heart%20disease%20cost%20the%20United,year%20from%202018%20to%202019.&text=This%20includes%20the%20cost%20of,lost%20productivity%20due%20to%20death

[9] Parker ED, Lin J, Mahoney T, . Economic costs of diabetes in the U.S. in 2022. Diabetes Care. 2024;47(1):26-43. doi:10.2337/dci23-0085 37909353

[10] Neubert L, König HH, Mietzner C, Brettschneider C. Dementia care-giving and employment: a mixed-studies review on a presumed conflict. Ageing Soc. 2021;41(5):1094-1125. doi:10.1017/S0144686X19001545

[11] Keita Fakeye MB, Samuel LJ, Drabo EF, Bandeen-Roche K, Wolff JL. Caregiving-related work productivity loss among employed family and other unpaid caregivers of older adults. Value Health. 2023;26(5):712-720. doi:10.1016/j.jval.2022.06.014 35973924 PMC9922792

[12] El-Hayek YH, Wiley RE, Khoury CP, . Tip of the iceberg: assessing the global socioeconomic costs of Alzheimer’s disease and related dementias and strategic implications for stakeholders. J Alzheimers Dis. 2019;70(2):323-341. doi:10.3233/JAD-190426 31256142 PMC6700654

[13] Aranda MP, Vega WA, Richardson JR, Resendez J. Priorities for optimizing health interventions across the life course in socially disadvantaged groups. Florida International University and UsAgainstAlzheimer’s. 2019. Accessed April 29, 2025. https://www.usagainstalzheimers.org/sites/default/files/2019-10/fiu_paper_10.18.19%20%281%29.pdf

[14] Manly JJ, Jones RN, Langa KM, . Estimating the prevalence of dementia and mild cognitive impairment in the US: the 2016 health and retirement study harmonized cognitive assessment protocol project. JAMA Neurol. 2022;79(12):1242-1249. doi:10.1001/jamaneurol.2022.3543 36279130 PMC9593315

[15] Daniel EV, Kleiman MJ, Galvin JE. Exploring reasons for differential vulnerability and Alzheimer’s disease risk in racial and ethnic minorities. J Alzheimers Dis. 2023;91(1):495-506. doi:10.3233/JAD-220959 36442203 PMC10515192

[16] Hinton L, Tran D, Peak K, Meyer OL, Quiñones AR. Mapping racial and ethnic healthcare disparities for persons living with dementia: a scoping review. Alzheimers Dement. 2024;20(4):3000-3020. doi:10.1002/alz.13612 38265164 PMC11032576

[17] Association of American Medical Colleges. 2022 Physician specialty data report. Accessed April 29, 2025. 2023. https://www.aamc.org/data-reports/workforce/report/physician-specialty-data-report

[18] Aranda MP, Kremer IN, Hinton L, . Impact of dementia: health disparities, population trends, care interventions, and economic costs. J Am Geriatr Soc. 2021;69(7):1774-1783. doi:10.1111/jgs.17345 34245588 PMC8608182

[19] National Equity Atlas. Data by ancestry shows wage differences among Latinos in the United States and selected high-cost metros. 2025. Accessed April 29, 2025. https://nationalequityatlas.org/data-in-action/stories/wage-gaps-by-latino-ancestry

[20] Gaskin DJ, Patrick R, LaVeist TA. The costs of Alzheimer’s and other dementia for African Americans. USAgainstAlzheimer’s. 2013. Accessed April 29, 2025. https://www.usagainstalzheimers.org/sites/default/files/USA2_AAN_CostsReport.pdf

[21] Wu S, Vega WA, Resendez J, Jin H. Latinos and Alzheimer’s disease: new numbers behind the crisis. UsAgainstAlzheimer’s. 2016. Accessed April 29, 2025. https://www.usagainstalzheimers.org/sites/default/files/Latinos-and-AD_USC_UsA2-Impact-Report.pdf

[22] Agency for Healthcare Research and Quality. Medical expenditure panel survey: survey background. 2023. Accessed April 29, 2025. https://meps.ahrq.gov/mepsweb/about_meps/survey_back.jsp 38416859

[23] Equator Network. STROBE Statement. Accessed April 29, 2025. https://www.equator-network.org/wp-content/uploads/2015/10/STROBE_checklist_v4_cross-sectional.pdf

[24] Ghosh K, Bondarenko I, Messer KL, . Attributing medical spending to conditions: a comparison of methods. PLoS One. 2020;15(8):e0237082. doi:10.1371/journal.pone.0237082 32776954 PMC7416958

[25] Gaskin, DJ, Richard, P, Walburn, J. The economical impact of pain. 2017. Accessed April 29, 2025. https://link.springer.com/chapter/10.1007/978-3-319-48046-6_1

[26] Aday LA, Andersen R. A framework for the study of access to medical care. Health Serv Res. 1974;9(3):208-220. 4436074 PMC1071804

[27] Grossman M. The Demand for Health: A Theoretical and Empirical Investigation. NBER Books; 1972.

[28] Deb P, Norton EC. Modeling health care expenditures and use. Annu Rev Public Health. 2018;39:489-505. doi:10.1146/annurev-publhealth-040617-013517 29328879

[29] Ettner SL. The impact of “parent care” on female labor supply decisions. Demography. 1995;32(1):63-80. doi:10.2307/2061897 7774731

[30] Becker G. Human Capital. 2nd ed. Columbia University Press; 1975.

[31] Donovan SA, Bradley DH. Real Wage Trends, 1979 to 2018. Congressional Research Service; 2019.

[32] Gianattasio KZ, Wu Q, Glymour MM, Power MC. Comparison of methods for algorithmic classification of dementia status in the Health and Retirement Study. Epidemiology. 2019;30(2):291-302. doi:10.1097/EDE.0000000000000945 30461528 PMC6369894

[33] Sanders GD, Neumann PJ, Basu A, . Recommendations for conduct, methodological practices, and reporting of cost-effectiveness analyses: second panel on cost-effectiveness in health and medicine. JAMA. 2016;316(10):1093-1103. doi:10.1001/jama.2016.12195 27623463

[34] Medical Expenditures Panel Survey. 2014-2020 Data files. Accessed May 2, 2025. https://meps.ahrq.gov/mepsweb/data_stats/download_data_files.jsp

[35] US Census Bureau. 2017 National population projections tables: main series. 2018. Accessed May 2, 2025. https://www.census.gov/data/tables/2017/demo/popproj/2017-summary-tables.html

[36] US Bureau of Labor Statistics. Consumer price index retroactive series (R-CPI-U-RS). 2023. Accessed December 11, 2023. https://www.bls.gov/cpi/research-series/r-cpi-u-rs-home.html

[37] Chidambaram P, Burns A. A look at nursing facility characteristics between 2015 and 2024. 2024. Accessed May 2, 2025. https://www.kff.org/medicaid/issue-brief/a-look-at-nursing-facility-characteristics/#:~:text=In%20order%20to%20receive%20payment,many%20deaths%20during%20the%20pandemic

[38] Harris-Kojetin L, Sengupta M, Lendon JP, Rome V, Valverde R, Caffrey C. Long-term care providers and services users in the United States, 2015–2016. National Center for Health Statistics. Accessed May 2, 2025. https://stacks.cdc.gov/view/cdc/76253 27023287

[39] Reckrey JM, Morrison RS, Boerner K, et al. Living in the community with dementia: who receives paid care?. J Am Geriatr Soc. 2020 Jan;68(1):186-191. doi:10.1111/jgs.16215 PMC6957088 31696511

[40] National Study of Caregiving. 2011-2017 data files. Accessed May 2, 2025. https://www.nhats.org/researcher/data-access

[41] US Bureau of Labor Statistics. Occupational employment and wages, May 2022: 31-1120 home health and personal care aides. 2023. Accessed May 8, 2023. https://www.bls.gov/oes/2022/may/oes311120.htm

[42] Panel Study of Income Dynamics. 2013 data file. Accessed May 2, 2025. https://simba.isr.umich.edu/DC/i.aspx

[43] IPUMS CPS. 1980-2019 data files. Accessed October 23, 2023. https://cps.ipums.org/cps-action/variables/group

[44] Internal Revenue Service. Statistics of income—2020 individual income tax returns. 2022. Accessed May 2, 2025. https://www.irs.gov/pub/irs-prior/p1304--2022.pdf

[45] Genworth. Genworth’s cost of care survey. 2023. Accessed October 29, 2024. https://www.carescout.com/cost-of-car

[46] Braga B, Butrica BA, Mudrazija S, Peters HE. Impacts of state paid family leave policies for older workers with spouses or parents in poor health. 2022. Accessed April 29, 2025. https://www.iza.org/publications/dp/15007/impacts-of-state-paid-family-leave-policies-for-older-workers-with-spouses-or-parents-in-poor-health

[47] Engage Family Caregiving Advisory Council and the Advisory Council to Support Grandparents Raising Grandchildren. Progress report: federal implementation of the 2022 national strategy to support family caregivers. 2024. Accessed April 29, 2025. https://acl.gov/sites/default/files/2024ProgressReport_StrategyToSupportCaregivers.pdf

[48] National Institute on Aging. National research summit on care, services, and supports for persons living with dementia and their care partners/caregivers. 2023. Accessed April 29, 2025. https://www.nia.nih.gov/sites/default/files/2023-07/caregiving-dementia-summit.pdf

[49] National Academies of Sciences, Engineering, and Medicine*. *Preventing and Treating Dementia: Research Priorities to Accelerate Progress. The National Academies Press; 2024. 39693470

