# Supplementary material for: Economic Burden of Alzheimer Disease and Related Dementias by Race and Ethnicity, 2020 to 2060
Source: JAMA Netw Open. 2025 Jun 5;8(6):e2513931. doi: 10.1001/jamanetworkopen.2025.13931 (PMC12142444; doi:10.1001/jamanetworkopen.2025.13931)
Supplement: Supplement 1. — eMethods. eTable 1. Summary Statistics for Dependents, Key Independent Variable, and Covariates Used in the Analysis eTable 2. Coefficients from Two-Part Models for Dependents, Key Independent Variable, and Covariates Used in the Analysis eTable 3. Estimated Differences in the Likelihood of Employment and Likelihood of Full-Time Employment Among Workers Between Caregivers and Matched Noncaregivers eTable 4. Matching Results for the Likelihood of Employment and Means and Variances of Raw and Matched Data for Noncollege Educated Dementia Caregivers and Noncaregivers eFigure. Ratio of Work-Related Costs to Value of Unpaid Care, 2020 to 2060 eTable 5. Current and Projected Costs of Paid Home Care and Nursing Home Care for Adults Living With ADRD Between 2020 and 2060 Assuming Stable Nursing Home Population at the 2020 Level, by Race and Ethnicity eReferences. [file jamanetwopen-e2513931-s001.pdf]

Supplemental Online Content

Mudrazija S, Aranda MP, Gaskin DJ, Monroe S, Richard P. Economic burden of Alzheimer disease and related dementias by race and ethnicity, 2020 to 2060. *JAMA Netw Open*. 2025;8(6):e2513931. doi:10.1001/jamanetworkopen.2025.13931

eMethods.

eTable 1. Summary Statistics for Dependents, Key Independent Variable, and Covariates Used in the Analysis

eTable 2. Coefficients from Two-Part Models for Dependents, Key Independent Variable, and Covariates Used in the Analysis

eTable 3. Estimated Differences in the Likelihood of Employment and Likelihood of Full-Time Employment Among Workers Between Caregivers and Matched Noncaregivers

eTable 4. Matching Results for the Likelihood of Employment and Means and Variances of Raw and Matched Data for Noncollege Educated Dementia Caregivers and Noncaregivers

eFigure. Ratio of Work-Related Costs to Value of Unpaid Care, 2020 to 2060

eTable 5. Current and Projected Costs of Paid Home Care and Nursing Home Care for Adults Living With ADRD Between 2020 and 2060 Assuming Stable Nursing Home Population at the 2020 Level, by Race and Ethnicity

eReferences.

This supplemental material has been provided by the authors to give readers additional information about their work.

© 2025 Mudrazija S et al. *JAMA Network Open*.

eMethods.

A. Estimating the Medical Care Costs and Work-Related Losses for Older Adults Living with ADRD

a. Data

We used data from the 2014-2020 Medical Expenditure Panel Survey (MEPS) to compute the medical care costs and work-related losses associated with Alzheimer's disease (ADRD) for African Americans and Latinos. The 2020 data from the MEPS was the most recent available at the time of the analysis. We pooled multiple years of data to increase the sample size. To our knowledge, MEPS is the sole data source that contains rich data on medical care costs and labor market outcomes, socio-demographic characteristics and a set of priority health conditions. Administered by the Agency for Healthcare Research and Quality (AHRQ), the MEPS is a nationally representative survey of the US civilian non-institutionalized population (AHRQ 2023).[1,2] For this analysis, we merged the Consolidated Household Component (HC) files with the Conditions files. The HC files contain socio-demographic characteristics, prevalence of health conditions for priority conditions and health status, insurance coverage, income, employment, healthcare utilization, and costs data on civilian non-institutionalized population in the United States. However, ADRD is not one of the priority health conditions that the MEPS collects data for. As a result, we merged the HC files with the health conditions files from 2014-2020 to capture ADRD and increase the sample size for the analysis of this condition. Conditions are measured in the Conditions files based on ICD-9 and ICD-10 codes. For population estimates, we used the same data sources from the Census Bureau that are used in the caregiving section of this report and that are explained in more detail there.

b. Study Populations and Design

For the medical care cost computations, we used data on the adult populations ages 50+. We divided ages into three categories: 50-64; 65-84 and 85+. The MEPS top coded adults who are 85 and older to protect data privacy for this population, especially for those who may have any rare health

© 2025 Mudrazija S et al. *JAMA Network Open*.

condition. For the productivity loss computations, we restricted the age groups to ages 50-64 and 65-70 to be able to capture the populations of elderly who actively participate in the labor force.

Our sample size for the medical spending analysis includes 31,028 individuals aged 50+ (representing a total population of 6,922,942 individuals). For the labor markets outcomes using data on individuals from ages 50 to 69, the sample size comprised of 3,458 individuals for the work hours analysis.

c. Measurement

There are multiple outcomes of interest in this analysis. First, direct medical care costs, which consist of payments made for hospital-based services, such as hospital inpatient services, emergency services, outpatient services (hospital, clinic, and office-based visits), prescription drugs, and other services (e.g., home health services, vision care services, ambulance services, dental care, and medical equipment). These payments also include out-of-pocket costs incurred by individuals as well as payments to health care providers made on their behalf by insurers, but they do not include health insurance premiums and over-the counter prescription drugs. The remaining outcomes of interest refer to lost productivity measure and include: 1) disability days; 2) annual hours worked; and 3) hourly wages forgone because of ADRD for adults aged between 50 and 70 years old. For this analysis, age was broken into two categories: 50-64 and 65-70. ADRD is the main condition of interest and was measured using the following ICD-9 codes: 290, 294 and 331 as well as ICD-10 codes of F01, F02, F03, G30 and R41.

d. Analytic Approach

Using the 2014-2020 MEPS data, we built regression models to estimate the annual direct medical care costs associated with ADRD compared to those without ADRD. This approach used the well-established incremental costs technique that compares persons with the health condition to those without the health condition (the counterfactuals).[3,4] We hypothesized that African Americans and Latinos with ADRD have higher incremental medical care costs compared to African Americans and Latinos without ADRD. To test our hypothesis, we selected the covariates or predictor variables based on the Aday and Andersen (1974) access to care conceptual model that is comprised.[5] Although this model

© 2025 Mudrazija S et al. *JAMA Network Open*.

represents the workhorse of health services researchers, it is important to note that there are strong similarities between this model, the Social Determinants of Health (SDOH) and the Grossman Model,[6] and all three models are widely used in the health economics literature.

Findings from this analysis should be easily replicable. Predisposing factors included age, race/ethnicity, and gender. Enabling factors were education, income, marital status, and health insurance status. The health needs factors included ADRD, self-reported general health status, diabetes, asthma, asthma attack, cancer, high blood pressure, heart attack, angina, other heart disease, emphysema/chronic bronchitis, joint pain, and arthritis. The final list of health conditions was selected after testing several model specifications. We also included census regions to capture variation in health insurance as well as time effects to capture any medical advances in the treatment of ADRD in the United States during 2014-2020. We used econometric techniques such as two-part regression models to compute medical care costs associated with ADRD.[7] These models allowed us to address several econometric challenges in the estimation of costs such as: non-negative values, the preponderance of zeros, skewness, and heteroskedastic errors where the variance of the error term is not constant. The first part of the model used logistic regression to predict whether individuals have any medical care costs and the second part of the model used generalized linear model (GLM) with log link and gamma expenditures to predict medical care costs given positive medical care costs. Further, we used a Park test (test for the relationship between mean and variance) to determine what specification to use and decided on a gamma specification for the medical care costs models. We used the survey regression procedure in STATA 18, which appropriately incorporates the complex design factors and sample weights. We conducted several specification and diagnostic tests of heteroskedasticity.[8] We used the CPI (Consumer Price Index) medical care index to adjust medical spending to the base year of 2020 and applied 3% annual inflation factor for the outyears.

For the lost productivity models, we hypothesized that African Americans and Latinos with ADRD experienced greater disability days, lower annual hours worked, and wages compared to African Americans and Latinos without ADRD. To estimate the loss labor market productivity models, we also used two-part models, similar to the medical care costs analysis. It is important to note that disability

© 2025 Mudrazija S et al. *JAMA Network Open*.

days, hours worked, and hourly wages are only observed for individuals who are working; hence, this may create potential selection bias as some individuals may not be working because of poor health conditions. To address issues of sample selection bias among those who are working and those who are not, the Heckman approach uses a distributional assumption, i.e., normality, to identify the likelihood of working in the labor market outcomes equations in the absence of instrumental variables. Labor markets findings from the two-part models have been found to be qualitatively equivalent to the Heckman approach when using distributional properties as an identification strategy.[9] That is, we used a logistic regression to estimate the probability of working in the first part of the model, and we used the second part to estimate hourly wages given that an individual is working. A similar approach was used to model disability days and annual hours worked. Based on Park test results we used a gamma distribution for the disability days models, a Gaussian distribution for the hours worked models and a gamma distribution for the hourly wages models. Specifications for these models were based on conceptual economic models of labor market participation and wages that use a Human Capital approach.[10] We controlled for demographic, socioeconomic, location, and health status and conditions characteristics as noted above. The only exception was that poverty levels were not included in the hourly wages equation as labor income was used to construct these poverty levels to avoid multicollinearity in the hourly wage models. Similar to medical care costs, we used the CPI index to adjust hourly wages to a base year of 2020 and assumed 3% annual growth in our projections.

B. Estimating the Value of Unpaid Care, Forgone Wages, Lost Labor Productivity and Other Costs for Caregivers of Older Adults Living with ADRD

a. Data

Data on family caregivers come from the 2011, 2015 and 2017 waves of the National Study of Caregiving (NSOC), a nationally representative supplement to the National Health and Aging Trends Study (NHATS), which collects information on family caregivers to Medicare beneficiaries aged 65 and older.[11] The survey includes data on the type, duration and intensity of help caregivers provided, possible effects of caregiving on caregivers, support services they may have used, and basic demographic and

© 2025 Mudrazija S et al. *JAMA Network Open*.

socioeconomic data, including work-related information. Caregivers are identified by the NHATS respondents during the interview and subsequently contacted. If a respondent identifies more than five caregivers, the survey randomly selects five caregivers for an interview. Sample weights account for survey non-response and differential probabilities of selection into the sample.

The NSOC analytic sample for this study includes Latino, African American, and non-Latino White[12] caregivers to NHATS respondents classified as likely having dementia from 2011-2017 waves of NSOC. Dementia classification in NHATS is determined based on an algorithm that utilizes three sources of relevant information: 1) self or proxy report of dementia diagnosis, 2) results of the AD8 Dementia Screening Interview, administered to proxy respondents, and 3) tests of memory, orientation, and executive function, administered to sample persons.[13]

While the NSOC was not originally designed as a longitudinal survey, because it elicits information on caregivers of the NHATS sample persons who are followed longitudinally, there is limited number of caregivers who provided information more than once. In such cases, we use information from the first wave when a caregiver participated in the survey.[14] Therefore, the analytic sample for this study has a pooled sample of 1,929 caregivers to persons with dementia, including 828 from the first wave, 653 from the second wave, and 448 from the third wave of NSOC. The sample has 1,116 non-Latino white, 644 non-Latino African American, and 169 Latino older adults. The NSOC data is supplemented with the relevant information on care recipients from the NHATS, including their age, race/ethnicity, dementia status, and financial transfers receipt.

Data on non-caregivers come from the 2013 wave of the Panel Study of Income Dynamics (PSID), a nationally representative longitudinal panel survey of US families. The key information on the provision of time support to parents comes from the 2013 Rosters and Transfers module and is supplemented with sociodemographic and health information from the main survey, including information on labor force status and hours worked.[15,16] The sample includes 8,615 heads and spouses who reported no provision of help to parents.[17]

© 2025 Mudrazija S et al. *JAMA Network Open*.

We additionally rely on data from multiple other sources to produce estimates of economic costs of providing care to older adults suffering from ADRD between 2016 and 2060. Projections of demographic trends by age and race and ethnicity come from the 2017 National Population Projections. Historic trends in educational attainment and household income by race and ethnicity are tracked using information from the Current Population Survey (CPS), available through IPUMS. We also use CPS for historical information on average wages. Consumer Price Index retroactive series (R-CPI-U-RS) information from the Bureau of Labor Statistics is used to adjust price levels over time to real (2020) U.S. dollars. Information on federal income tax payments by adjusted gross income levels for tax year 2020 (filed in 2021) comes from the Internal Revenue Service. Data on home health and personal care aides' mean hourly wage as of 2022 comes from the Bureau of Labor Statistics.

b. Analytic Approach

The analysis begins with an overview of the current value of unpaid care, lost wages and productivity, loss of federal income tax revenue, and financial transfers from unpaid caregivers. To make these estimates, we perform Mahalonobis (multivariate-distance) matching of caregivers to older adults from NSOC with their non-caregiver peers from PSID, stratifying the matching by race and ethnicity and requesting exact match with respect to caregivers' gender and marital status. The samples are matched on the following sociodemographic and health characteristics that were comparable across the two surveys: age (in years), gender, marital status (married vs. unmarried), education attainment (college degree or higher vs. less than a college degree), homeownership, self-rated overall health status (excellent/very good, good, and fair/poor), and a binary predictor for any living siblings. These results are used alongside information from other data sources to derive current period estimates of economic costs of providing care to persons with ADRD.

Next, the matching is repeated by college degree attainment for each of the three major racial/ethnic groups of interest to accommodate for the historic trend of growth in educational attainment, which has continued and even accelerate in recent years, and shows no sign of stopping or reversing. Alongside the information from other available resources, this allows us to derive estimates of various

© 2025 Mudrazija S et al. *JAMA Network Open*.

cost categories for the population of interest. Accounting for educational attainment change over time in addition to changes in racial and ethnic composition of the population is important given that the opportunity cost of caregiving is much higher for those with at least a college degree, since they are both much more likely to work and, on average, earn much higher hourly wage than those without a college degree.

Accounting explicitly for future changes in educational attainment is critical inasmuch recent research shows divergent trends in real wages for workers with different educational attainment (Donovan and Bradley 2018). Furthermore, differences in economic costs of caregiving by educational attainment vary by race and ethnicity. With such trends and inherent uncertainty, we test several alternative scenarios for the expected change in educational attainment beyond 2020 and effectively establish a range of estimates for the opportunity cost of unpaid caregiving through 2060. While the projections presented in the paper are based on average gains in educational attainment since 2000, we also calculated versions with lower projected educational gains (based on longer-term average gains dating back to 1976) and higher projected gains (based on the most recent trends, with 2010 as the initial year). These results are available on request.

Furthermore, we account for differential trends in real wage growth by educational attainment. Using data from the Current Population Survey, we calculate that between 1999 and 2018 (i.e., over the years preceding the onset of the Covid-19 pandemic), real wages increased by a total of 4.3% for those with less than a college degree, and 8.3% for those with at least a college degree. We extrapolate these real wages growth rates into the future. Similarly, we account for differential trends in household income by race and ethnicity to use for calculating the relative burden of forgone earnings as a percentage of household income and extrapolate pre-pandemic trends into the future years. We calculate that the household income increased by 16.4% for Latinos, 11.2% for non-Latino Whites, and 3.1% for African Americans between 1999 and 2018. Consistent with the estimates for older adults with dementia, we adjust the estimates of future economic impacts associated with caregiving for them by 3% annually

© 2025 Mudrazija S et al. *JAMA Network Open*.

starting with 2020 as the base year. Therefore, our projections are expressed in what could be considered nominal dollars assuming the 3-percent long-term trend of increase in consumer prices.

We make several simplifying assumptions that allow future estimates to be derived. First, we assume that the overall provision of care will increase proportionately with the increase in the ADRD population with LTSS needs. Therefore, unmet care needs will not change. Second, we assume unchanged preference for informal caregiving, disregarding the possibility that the relative prices of alternatives to informal caregiving—primarily paid home care and nursing homes—may change and thereby shift the demand for informal caregiving independently of population characteristics. If such a change would occur, the estimate could be either too high (if the relative price of alternatives declines) or too low (if it increases). Furthermore, we assume that the relative prices of family and paid care will remain broadly constant, and that the profiles of care recipients for each subgroup of caregivers will remain constant over time.

C. Estimating the Costs of Formal Care for Older Adults Living with ADRD

a. Data

Data on the current costs of nursing home care and in-home paid care come from the Genworth's Cost of Care Survey.[18] This survey provides per person cost of different types of formal care as well as projections of these costs through 2060, applying the same 3% inflation factor that we use in our calculations. These data are supplemented with the current and historic information on nursing home population from the Kaiser Family Foundation,[19] as well as with data on the share of nursing home population by race and ethnicity from the Centers for Disease Control and Prevention,[20] and information on the prevalence and intensity of paid community care is from a 2020 article by Reckrey and colleagues.[21] Similar to other estimate included in this manuscript, we use Census Bureau population projections to account for population growth and compositional changes through 2060.

Analytic Approach

Utilizing the information from the Genworth’s Cost of Care Survey alongside that from other data sources as described, we calculate the cost of formal care. Our default assumption is that the use of formal

© 2025 Mudrazija S et al. *JAMA Network Open*.

care services will increase commensurately with the increase in the older adult population, same as the increase in the utilization of unpaid family care. In the alternative calculation, we assume that nursing home population remains flat at the 2020 level due to continued efforts to keep older adults out of the institutional care and in the community, and that a part of these older adults "diverted" from nursing homes will receive paid home care. For all the calculations, we assume that those who report receiving less than 20 hours of paid weekly care (58% of the sample), receive 10 hours of care per week, while those reporting receiving 20 hours or more of care per week (42% of the sample), are assigned a value of 20 hours of paid care. Furthermore, we assume that three quarters of all nursing home residents live in semi-private and one quarter in private rooms, and adjust average nursing home costs accordingly.

Additional Results

1. **Descriptive and Inferential Results Used in Calculations of Economic Impact for Older Adults Living with ADRD**

eTable 1. Summary Statistics for Dependents, Key Independent Variable, and Covariates Used in the Analysis

	Medical Care Costs<br>Mean<br>95% CI	Hours Worked<br>Mean<br>95% CI
Any Expenditures	0.97<br>[0.97,0.97]	
Total Expenditures	10715.32<br>[10378.83,11051.81]	
ADRD	0.01<br>[0.00,0.00]	0.007<br>[-0.00,0.00]
Fair/Poor Health	0.18<br>[0.17,0.18]	0.15<br>[0.14,0.17]
Hypertension	0.56<br>[0.55,0.57]	0.47<br>[0.45,0.49]
High Cholesterol	0.53<br>[0.52,0.54]	0.45<br>[0.44,0.47]
Diabetes	0.20<br>[0.19,0.20]	0.17<br>[0.16,0.19]
Coronary Disease/Angina	0.10<br>[0.10,0.11]	0.08<br>[0.07,0.09]
Asthma	0.08<br>[0.07,0.08]	0.09<br>[0.08,0.10]

© 2025 Mudrazija S et al. *JAMA Network Open*.

	Medical Care Costs<br>Mean<br>95% CI	Hours Worked<br>Mean<br>95% CI
Emphysema/Chronic Broch	0.05<br>[0.04,0.05]	0.05<br>[0.04,0.06]
Joint Pain/Arthritis	0.31<br>[0.30,0.31]	0.24<br>[0.22,0.26]
Cancer	0.17<br>[0.17,0.18]	0.13<br>[0.12,0.14]
Female	0.54<br>[0.53,0.54]	0.53<br>[0.52,0.55]
Male	0.46<br>[0.46,0.47]	0.47<br>[0.45,0.48]
Age 45-54	0.17<br>[0.17,0.18]	0.25<br>[0.23,0.27]
Age 55-64	0.35<br>[0.35,0.36]	0.52<br>[0.50,0.55]
Age 65-74	0.28<br>[0.27,0.28]	0.22<br>[0.21,0.24]
Age>75	0.20<br>[0.19,0.20]	0.00<br>[0.00,0.00]
White	0.75<br>[0.73,0.76]	0.72<br>[0.69,0.74]
African American	0.10<br>[0.09,0.11]	0.11<br>[0.10,0.13]
Latino	0.10<br>[0.09,0.11]	0.11<br>[0.10,0.13]
Asian	0.03<br>[0.03,0.03]	0.03<br>[0.02,0.05]
Native American	0.00<br>[0.00,0.01]	0.01<br>[0.00,0.01]
API	0.02<br>[0.01,0.02]	0.02<br>[0.01,0.02]
Married	0.61<br>[0.60,0.62]	0.64<br>[0.62,0.67]
Divorced	0.17<br>[0.16,0.17]	0.19<br>[0.17,0.20]
Separate/Widowed/Never Married	0.22<br>[0.21,0.23]	0.17<br>[0.15,0.19]
No High School	0.12<br>[0.11,0.12]	0.09<br>[0.08,0.11]
High School	0.29<br>[0.28,0.30]	0.27<br>[0.25,0.29]
Some College/Associate Degree	0.25<br>[0.24,0.26]	0.27<br>[0.25,0.30]
College or More	0.21<br>[0.20,0.22]	0.36<br>[0.33,0.38]
Near/Below Poverty	0.04<br>[0.04,0.04]	0.03<br>[0.03,0.04]
Low Income	0.12<br>[0.11,0.12]	0.10<br>[0.08,0.11]

© 2025 Mudrazija S et al. *JAMA Network Open*.

	Medical Care Costs<br>Mean<br>95% CI	Hours Worked<br>Mean<br>95% CI
Middle Income	0.26	0.24
	[0.25,0.27]	[0.22,0.26]
Private Health Insurance	0.59	0.70
	[0.58,0.60]	[0.68,0.72]
Public Health Insurance	0.32	0.21
	[0.31,0.33]	[0.19,0.23]
No Health Insurance	0.03	0.04
	[0.03,0.04]	[0.04,0.05]
Northeast	0.18	0.18
	[0.17,0.20]	[0.15,0.21]
Midwest	0.22	0.21
	[0.20,0.23]	[0.18,0.23]
South	0.38	0.38
	[0.36,0.40]	[0.34,0.42]
West	0.22	0.23
	[0.21,0.24]	[0.20,0.26]
Year 1	0.03	0.00
	[0.03,0.04]	[0.00,0.00]
Year 2	0.15	0.00
	[0.14,0.16]	[0.00,0.00]
Year 3	0.27	0.00
	[0.26,0.28]	[0.00,0.00]
Year 4	0.16	1.00
	[0.16,0.17]	[1.00,1.00]
Year 5	0.12	0.00
	[0.11,0.12]	[0.00,0.00]
Year 6	0.26	0.00
	[0.25,0.28]	[0.00,0.00]
Employment Status		0.64
		[0.62,0.66]
Ann Work Hours		1299.52
		[1251.67,1347.37]
N	31,028	3,458

Source: MEPS, 2014-2020

eTable 2. Coefficients from Two-Part Models for Dependents, Key Independent Variable, and Covariates Used in the Analysis

Variables	Total Expenditures (N=31,028)<br>b/se		Annual Work Hours (N=3,458)<br>b/se	
	Logistics	GLM	Logistics	GLM
ADRD	-0.80	0.31**	0.21	-0.08
	(0.58)	(0.14)	(0.58)	(0.14)
Fair/Poor Health	0.47***	0.72***	-1.00***	-0.01
	(0.12)	(0.03)	(0.05)	(0.01)
Hypertension	0.98***	0.20***	-0.17***	0.00
	(0.10)	(0.03)	(0.05)	(0.01)

© 2025 Mudrazija S et al. *JAMA Network Open*.

High Cholesterol	0.90***	0.05	-0.09*	-0.01
	(0.10)	(0.03)	(0.05)	(0.01)
Diabetes	1.12***	0.37***	-0.19***	0.00
	(0.18)	(0.03)	(0.06)	(0.01)
Coronary Disease/Angina	0.17	0.35***	-0.34***	-0.03*
	(0.27)	(0.04)	(0.09)	(0.02)
Asthma	0.77***	0.26***	-0.17*	0.01
	(0.25)	(0.05)	(0.09)	(0.02)
Emphysema/Chronic Broch	0.87**	0.20***	-0.57***	-0.06**
	(0.34)	(0.06)	(0.12)	(0.03)
Joint Pain/Arthritis	0.21*	0.07*	-0.03	-0.01*
	(0.11)	(0.04)	(0.05)	(0.01)
Cancer	0.63***	0.44***	-0.25***	-0.02
	(0.17)	(0.04)	(0.07)	(0.01)
Female	0.62***	0.04	-0.54***	-0.13***
	(0.08)	(0.03)	(0.05)	(0.01)
Age 45-54	-0.96***	-0.29***	1.89***	0.16***
	(0.20)	(0.06)	(0.07)	(0.01)
Age 55-64	-0.93***	-0.19***	1.09***	0.12***
	(0.20)	(0.04)	(0.06)	(0.01)
Age 65-74	-0.44**	-0.15***		
	(0.21)	(0.04)		
White	-0.52	0.21	1.20*	0.19
	(0.88)	(0.49)	(0.67)	(0.13)
African American	-1.07	0.11	1.14*	0.20
	(0.89)	(0.49)	(0.68)	(0.13)
Latino	-0.98	-0.07	1.60**	0.20
	(0.87)	(0.48)	(0.67)	(0.13)
Asian	-1.23	-0.37	1.46**	0.21
	(0.90)	(0.49)	(0.69)	(0.13)
Native American	-0.19	0.55	0.80	0.14***
	(0.63)	(0.43)	(0.54)	(0.04)
API	-1.51*	-0.42	1.16	0.22*
	(0.91)	(0.49)	(0.71)	(0.13)
Married	0.26**	-0.12***	-0.17***	0.01
	(0.11)	(0.03)	(0.06)	(0.01)
Divorced	-0.00	-0.03	0.35***	0.04***
	(0.13)	(0.04)	(0.07)	(0.01)
No High School	-0.66***	-0.09	-0.52***	0.03*
	(0.20)	(0.06)	(0.09)	(0.02)
High School	-0.44***	-0.07	-0.22***	0.02*
	(0.16)	(0.05)	(0.08)	(0.01)
Some College/Associate Degree	-0.15	-0.00	-0.13	-0.01
	(0.17)	(0.05)	(0.08)	(0.01)
College or More	0.18	0.06	0.13	0.00
	(0.21)	(0.05)	(0.09)	(0.01)
Near/Below Poverty	-0.34*	0.05	-0.67***	-0.11***
	(0.20)	(0.06)	(0.12)	(0.03)
Low Income	-0.17	-0.02	-0.42***	-0.08***
	(0.12)	(0.05)	(0.07)	(0.01)
Middle Income	-0.13	-0.07**	-0.05	-0.04***

© 2025 Mudrazija S et al. *JAMA Network Open*.

	(0.11)	(0.03)	(0.05)	(0.01)
Public Health Insurance	0.12	0.02	-0.82***	-0.17***
	(0.21)	(0.05)	(0.10)	(0.02)
No Health Insurance	-1.36***	-0.61***	0.43***	-0.01
	(0.24)	(0.14)	(0.13)	(0.03)
Northeast	-0.06	0.04	0.20**	0.00
	(0.17)	(0.05)	(0.08)	(0.01)
Midwest	0.01	0.02	0.01	0.02**
	(0.15)	(0.04)	(0.08)	(0.01)
South	-0.10	-0.09**	-0.09	0.03***
	(0.13)	(0.04)	(0.08)	(0.01)
Year 2	-1.17***	-0.38***	0.25*	0.02
	(0.20)	(0.10)	(0.13)	(0.02)
Year 3	-0.89***	0.01	-0.11	-0.01
	(0.15)	(0.05)	(0.07)	(0.01)
Year 4	-0.54***	-0.09**	0.03	-0.01
	(0.14)	(0.04)	(0.07)	(0.01)
Year 5	0.89***	0.03	-0.03	-0.02
	(0.22)	(0.05)	(0.07)	(0.01)
Year 6	-0.57***	0.03	0.19***	-0.01
	(0.14)	(0.06)	(0.07)	(0.01)
Year 7	0.00	0.00	0.00	0.00
	(.)	(.)	(.)	(.)
Constant	3.85***	8.76***	-0.94	7.26***
	(0.92)	(0.49)	(0.72)	(0.13)

Note: * p<.10, ** p<0.05, *** p<.01
Source: MEPS, 2014-2020

2. Example of Matching Results Used in Calculations of Economic Impact for Older Adults Living with ADRD

Here, we aim to give an insight into the results of our matching procedure and the quality of information that we then utilize in calculating employment-related costs of caregiving. In Table A3 we show the full set of estimates of differences between caregivers and matched non-caregivers in the likelihood of employment and likelihood of full-time employment. The results suggest that caregiving is related with a noticeable decline in the likelihood of employment and full-time employment among caregivers relative to non-caregivers. For the likelihood of employment, having a college degree is associated with a larger difference in the likelihood of employment, especially for Latino adults and, somewhat less so, African American adults. For the full-time employment, we find divergent trends by

© 2025 Mudrazija S et al. *JAMA Network Open*.

race and ethnicity, with larger differences in the likelihood for college educated minoritized groups and non-college educated White adults.

eTable 3. Estimated Differences in the Likelihood of Employment and Likelihood of Full-Time Employment Among Workers Between Caregivers and Matched Noncaregivers

	**No college degree**		
	African American	Latino	White
Difference in the likelihood of employment	-0.077	-0.106	-0.085
Difference in the likelihood of full-time employment among workers	-0.095	-0.159	-0.108

	**College degree**		
	African American	Latino	White
Difference in the likelihood of employment	-0.155	-0.334	-0.100
Difference in the likelihood of full-time employment among workers	-0.161	-0.196	-0.073

Source: National Study of Caregiving, 2011-2017; National Health and Aging Trends Study, 2011-2017; Panel Study of Income Dynamics, 2013; authors' estimates.

Each row of estimates in table A3 is a result of a separately fitted matching procedure. To illustrate the mechanics of it, in table A4 we further present results for one of these estimates, for the likelihood of employment for non-college educated dementia caregivers.

eTable 4. Matching Results for the Likelihood of Employment and Means and Variances of Raw and Matched Data for Noncollege Educated Dementia Caregivers and Noncaregivers

Matching results					
	Treated		Controls		Bandwidth
	Yes	No	Yes	No	
**Matched sample size**					
African American	484	12	2032	42	1.703
Latino	141	0	636	0	3.524
White	697	23	2147	428	0.84

	Coefficient	Standard error	t value	P>t	95% confidence interval
**ATT**					
African American	-0.077	0.034	-2.29	0.022	[-0.142 - -0.011]
Latino	-0.106	0.05	-2.1	0.035	[-0.205 - -0.007]
White	-0.085	0.031	-2.71	0.007	[-0.146 - -0.023]

Means and variances of raw and matched data								
	Raw data				Matched data			
	Treated		Controls		Treated		Controls	
	Mean	Variance	Mean	Variance	Mean	Variance	Mean	Variance
**African American**								
Age (in years)	57.8	196.1	45.7	258.5	57.8	187.6	55.0	212.9
Female	0.69	0.21	0.58	0.24	0.69	0.22	0.69	0.22
Married/partnered	0.42	0.24	0.42	0.24	0.42	0.24	0.42	0.24
Homeownership	0.48	0.25	0.37	0.23	0.47	0.25	0.47	0.25
Self-rated health (ref. Excellent/Very good)								
Good	0.30	0.21	0.34	0.22	0.31	0.21	0.31	0.21
Fair/Poor	0.27	0.20	0.26	0.19	0.27	0.20	0.27	0.20
Any living siblings	0.54	0.25	0.91	0.08	0.55	0.25	0.55	0.25
**Latino**								
Age (in years)	53.6	220.7	45.0	244.3	53.6	220.7	47.2	259.7
Female	0.69	0.22	0.56	0.25	0.69	0.22	0.69	0.22
Married/partnered	0.54	0.25	0.73	0.20	0.54	0.25	0.54	0.25
Homeownership	0.45	0.25	0.47	0.25	0.45	0.25	0.41	0.24
Self-rated health (ref. Excellent/Very good)								
Good	0.38	0.24	0.35	0.23	0.38	0.24	0.40	0.24
Fair/Poor	0.23	0.18	0.28	0.20	0.23	0.18	0.25	0.19
Any living siblings	0.65	0.23	0.94	0.06	0.65	0.23	0.81	0.16
**White**								
Age (in years)	62.2	201.7	52.1	344.2	61.8	195.3	61.1	197.2
Female	0.68	0.22	0.54	0.25	0.69	0.21	0.69	0.21
Married/partnered	0.72	0.20	0.69	0.22	0.73	0.20	0.73	0.20
Homeownership	0.73	0.20	0.66	0.22	0.75	0.19	0.75	0.19
Self-rated health (ref. Excellent/Very good)								
Good	0.29	0.21	0.32	0.22	0.29	0.21	0.29	0.21
Fair/Poor	0.20	0.16	0.21	0.17	0.20	0.16	0.20	0.16
Any living siblings	0.54	0.25	0.91	0.08	0.56	0.25	0.56	0.25

Source: National Study of Caregiving, 2011-2017; National Health and Aging Trends Study, 2011-2017; Panel Study of Income Dynamics, 2013; authors' estimates.

The results show that there are between three and five PSID matched non-caregivers (i.e., controls) used for every NSOC caregiver (i.e., treated). Very small proportion (1.8%) of caregivers (or 25 out of 1,347) were not used because of the lack of appropriate matching controls. Following the matching

© 2025 Mudrazija S et al. *JAMA Network Open*.

procedure, we get a matched sample that has a much more similar profile than before the matching, with only some residual difference in the average age. All other matching results are available on request.

3. Relative Increase in Work-Related Costs and Value of Unpaid Care

Although historically the value of unpaid (family) care has been larger than the value of forgone earnings that caregivers could have earned had they worked and lower productivity among working caregivers, our projections suggest that these trends are changing and may even reverse in the relatively near future. Across all caregivers, the work-related costs of caregiving, which include foregone wages and productivity loss, are increasing at a faster pace than the value of unpaid family care. Figure A1 suggests that by 2060 these work-related costs will be more than two thirds as high (if caregiving valued at replacement wage) and 42% higher (if valued at minimum wage) than the value of unpaid family care. These results are important inasmuch they suggest that it is becoming increasingly important to support continued labor force attachment of caregivers to minimize the adverse economic impact that providing care to loved ones can have both for their family finances and broader economy.

eFigure. Ratio of Work-Related Costs to Value of Unpaid Care, 2020 to 2060

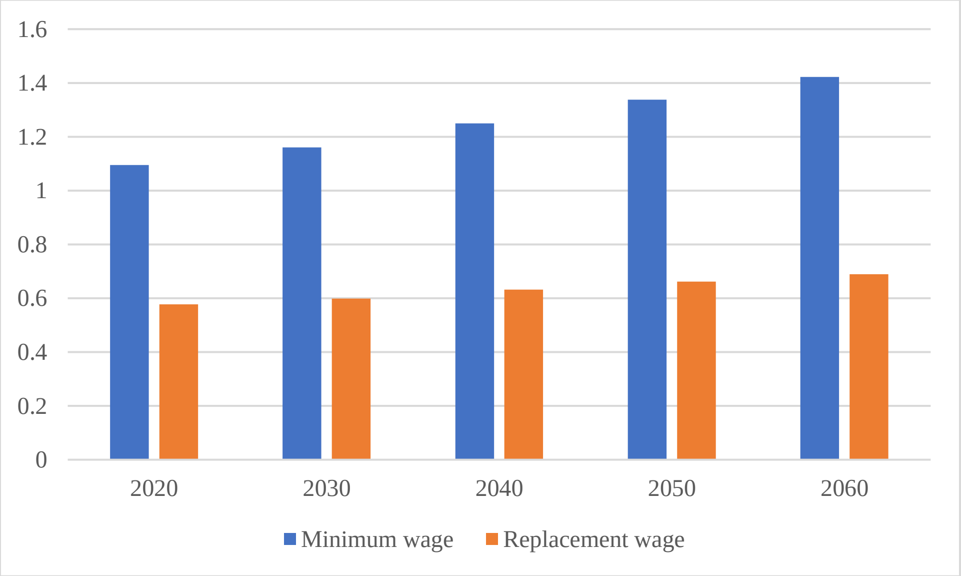

Note: Actual (2020) and projected (2030, 2040, 2050, and 2060) amounts are based on the population numbers from the Census Bureau; estimates are adjusted annually by 3% using 2020 as the baseline year.

© 2025 Mudrazija S et al. *JAMA Network Open*.

4. Approximations of Formal Care Costs Using Alternative Assumptions

Our approximations of formal care costs represent just one possible scenario of future costs that critically depends on the assumption of the growth in future use of nursing homes and paid home care commensurate with older adult population growth. However, recent history has witnessed essentially no growth in nursing home population despite population aging as the policy efforts have been aimed at rebalancing long-term services and supports from institutionalization to aging in place. Therefore, in our alternative scenario, presented in Table A5, we make calculations assuming continuously stable nursing home population at the 2020 level and an additional increase in population receiving paid home care commensurate with their current share of older adults receiving care in the community. These results suggest that the growth in formal care costs would be substantially slower, driven primarily by the assumption that unpaid family caregivers would provide exclusive care for almost three quarter of these older adults, but also because of lower costs of paid home care relative to nursing home care for the remaining one quarter of older adults who would otherwise be institutionalized. In 2060, for example, the difference in formal care costs would exceed $200 billion. However, the overall “savings” of such redistribution cannot be assumed as they fail to account for increased family caregiving burden and related financial and nonfinancial costs. Moreover, because individuals in nursing homes may require more intensive care, it is likely reasonable to expect that paid home care for this population would be substantially more common than for other older adults needing care who live in the community, which would further decrease the difference between our original and alternative estimates. Yet, we still consider the two estimates useful to be considered jointly as upper and lower bound approximations of possible future costs of paid care for older adults with ADRD by race and ethnicity.

eTable 5. Current and Projected Costs of Paid Home Care and Nursing Home Care for Adults Living With ADRD Between 2020 and 2060 Assuming Stable Nursing Home Population at the 2020 Level, by Race and Ethnicity

	2020	2030	2040	2050	2060
**Paid home care***					

© 2025 Mudrazija S et al. *JAMA Network Open*.

African American	3.4	10.3	19.2	33.0	52.3
Latino	3.7	13.0	26.8	50.1	86.5
White	18.7	45.1	74.1	107.6	139.8
**Nursing home care****					
African American	8.7	14.0	20.5	30.6	46.2
Latino	3.3	6.3	10.3	16.8	27.6
White	45.6	58.6	75.3	95.1	117.6
**Total**	83.3	147.2	226.1	333.2	470.1

Notes: * Assuming average of 10 hours of weekly care for those (58%) reporting receiving less than 20 hours of care and 30 hours of care for those (42%) reporting receiving more than 20 hours of care.
** Assuming 3/4 of all nursing home residents live in semi-private and 1/4 in private rooms.

Beyond the assumptions related to future nursing home use, we recognize the uncertainty related to other assumptions we make in this paper. We tested the sensitivity of several such assumptions, such as selecting different reference period for calculating average gains in college education. These results are available to readers on request.

© 2025 Mudrazija S et al. *JAMA Network Open*.

eReferences

[1] Agency for Healthcare Research and Quality. *Medical Expenditure Panel Survey: survey background*. 2023. Retrieved from https://meps.ahrq.gov/mepsweb/about_meps/survey_back.jsp.
[2] ibid
[3] Ghosh K, Bondarenko I, Messer KL, Stewart ST, Raghunathan T, Rosen AB, Cutler DM. Attributing medical spending to conditions: a comparison of methods. PLoS One. 2020;15(8):e0237082.
[4] Gaskin, DJ, Richard, P, Walburn, J. The economical impact of pain. *Neuroimaging of Pain*, 2017; 1-17.
[5] Aday LA, Andersen R. A framework for the study of access to medical care. *Health Serv Res*. 1974; Fall;9(3):208-20.
[6] Grossman, M. *The demand for health: a theoretical and empirical investigation*. 1972. NBER Books.
[7].Deb, P, Norton, EC. Modeling health care expenditures and use. *Annual Review of Public Health*, 2018;*39*:489-505.
[8]ibid
[9] Ettner, S. L. (1995). The impact of "parent care" on female labor supply decisions. *Demography*, 1995; *32*(1):63-80.
[10] Becker, G. *Human capital* (2nd ed). 1975. New York, NY: Columbia University Press.
[11] Medicare covers all adults aged 65 and older (as well as people with disabilities), except those who did not meet the program's work requirement and are not married to someone who met the work requirement, and foreign-born people who are neither US citizens nor permanent residents.
[12] Although the key focus of our analysis is Latino and African American population of older adults with dementia and their caregivers, we utilize information on their non-Hispanic white peers when needed for comparative purposes.
[13] More details available in: Kasper JD, Freedman VA, Spillman BC. *Classification of persons by dementia status in the National Health and Aging Trends Study*. Technical paper. 2013 May; 5:1-14.
[14] Starting in 2017, NSOC started with a longitudinal component, which collected interviews from caregivers originally identified in 2015. However, for this study, we focus strictly on the "cross-sectional" samples of NSOC across the three waves of interest, including 2011, 2015, and 2017.
[15] The reference periods in the two surveys are somewhat different. While the NSOC asks about working in the prior week, PSID asks about working at the time of the interview. Similarly, the NSOC asks about hours worked in the prior week, whereas PSID asks about average hours worked in the prior year. Given that the NSOC does not make any explicit reference to overtime hours, the analysis in this study relies on a more conservative PSID measure of hours worked that leaves out any overtime hours worked.
[16] While the design of the PSID questionnaire allows identifying non-helpers, it is not appropriate for identifying helpers to parents (or other family members) for health or functioning reasons as it does not distinguish this type of help from other types of practical help. Therefore, it is not possible to analyze work-related impacts of caregiving using the PSID data only.
[17] Because the PSID questionnaire elicits information on the provision of time support to parents only, it is possible that some persons in the non-caregiver sample provide care to others. However, as about three-quarters of working-age caregivers to older adults are children and the prevalence of caregiving for this population is below 7%, no more than 2% of the non-caregiver sample might be affected. Given the observed negative impact of caregiving on the likelihood of working and hours worked, this could result in a marginal downward bias of the estimates.
[18] Detailed information available at https://www.genworth.com/aging-and-you/finances/cost-of-care.
[19] For details, see https://www.kff.org/medicaid/issue-brief/a-look-at-nursing-facility-characteristics/#:~:text=In%20order%20to%20receive%20payment,many%20deaths%20during%20the%20pandemic.
[20] More details available in: Harris-Kojetin L, Sengupta M, Lendon JP, Rome V, Valverde R, Caffrey C. Long-term care providers and services users in the United States, 2015–2016. National Center for Health Statistics. Vital Health Stat 2019; 3(43)
[21] Reckrey JM, Morrison RS, Boerner K, Szanton SL, Bollens-Lund E, Leff B, Ornstein KA. Living in the community with dementia: Who receives paid care?. *Journal of the American Geriatrics Society*. 2020 Jan;68(1):186-91.

© 2025 Mudrazija S et al. *JAMA Network Open*.
