# Supplementary material for: Economic Burden of Alzheimer Disease and Related Dementias by Race and Ethnicity, 2020 to 2060
Source: JAMA Netw Open. 2025 Jun 5;8(6):e2513931. doi: 10.1001/jamanetworkopen.2025.13931 (PMC12142444; doi:10.1001/jamanetworkopen.2025.13931)
Supplement: Supplement 2. — Data Sharing Statement [file jamanetwopen-e2513931-s002.pdf]

Data Sharing Statement

Mudrazija. Economic Burden of Alzheimer Disease and Related Dementias by Race and Ethnicity, 2020 to 2060. *JAMA Netw Open*. Published June 05, 2025. doi:10.1001/jamanetworkopen.2025.13931

Data
Data available: No

Additional Information
Explanation for why data not available: We use secondary data sources that are publicly available to all users per rules and regulations set by the institutions producing and storing these data. Accessing these data generally requires each user to register and agree to follow the data use policies prior to being granted permission to use the data.
